# mRNA-LNP vaccine providing antigen and co-stimulation in the tumor microenvironment enhances CAR T cell function (CART-Vac)

**DOI:** 10.1016/j.omton.2026.201234

**Published:** 2026-05-14

**Authors:** Ikumi Nakashima, Shoji Saito, Jingbo Zhao, Miyuki Tanaka, Eiichi Akahoshi, Aiko Hasegawa, Mitsuko Sugano-Ishihara, Shigeki Yagyu, Yozo Nakazawa

**Affiliations:** 1 Department of Pediatrics, Shinshu University School of Medicine, Asahi 3-1-1, Matsumoto, Nagano 390-8621, Japan; 2 Center for Advanced Research of Gene and Cell Therapy, Shinshu University, Matsumoto, Japan; 3 Corporate Laboratory, Toshiba Corporation, Komukai-Toshiba 1, Saiwai-Ku, Kawasaki, Kanagawa 212-8582, Japan; 4 Innovative Research and Liaison Organization, Shinshu University, Asahi 3-1-1, Matsumoto, Nagano 390-8621, Japan

**Keywords:** chimeric antigen receptor, co-stimulatory molecules, gene therapy, intratumor injection, lipid nanoparticles, mRNA, target antigens, rhabdomyosarcoma, vaccine, EPHB4

## Abstract

Despite advances in multimodal therapies, outcomes for pediatric patients with relapsed or refractory cancers remain poor. Chimeric antigen receptor (CAR) T cell therapy has demonstrated limited efficacy in solid tumors due to the immunosuppressive tumor microenvironment (TME), which promotes T cell exhaustion and restricts CAR T cell expansion. This study evaluated a combinatorial approach to enhance CAR T function by reprogramming the TME to overexpress target antigens (TAs) and co-stimulatory molecules (CSMs) through a lipid nanoparticle-based CAR-T vaccination (CART-Vac). As proof of concept, rhabdomyosarcoma cells (Rh30) engineered to overexpress TAs and CSMs (Rh30-TACS) were examined. EPHB4-directed CAR T cells demonstrated enhanced cytotoxicity, proliferation, and cytokine secretion *in vitro*, and superior tumor control with increased T cell infiltration in Rh30-TACS tumors *in vivo* compared with Rh30 tumors. To induce TA and CSM expression in the TME, CART-Vac was designed to deliver mRNAs encoding truncated EPHB4, CD80, and CD137L. CART-Vac effectively mediated transient expression, significantly enhancing CAR T expansion and antitumor activity in both models. These findings suggest that CART-Vac can modulate the TME, offering a promising strategy to improve the therapeutic efficacy of CAR T cells in solid tumors.

## Graphical abstract

**Figure:**
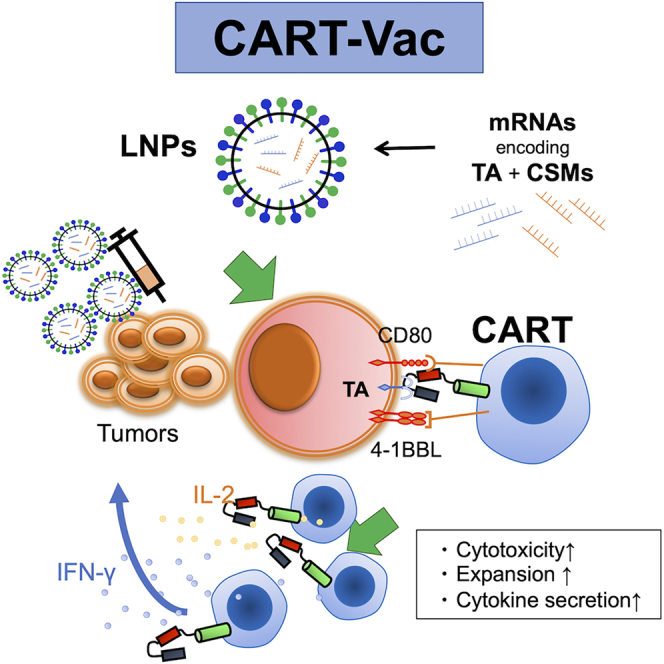

## Teaser

Saito and colleagues propose a novel strategy (CART-Vac) that employs tumor-tropic LNPs to reprogram the tumor microenvironment through TA and CSM overexpression. This approach significantly enhances CAR T cell cytotoxicity, expansion, and Th1 cytokine secretion, resulting in improved anti-tumor effects against solid tumors.

## Introduction

Although recent advances in multimodal therapies have improved outcomes in pediatric cancers, the prognosis for relapsed or refractory (R/R) cases remains poor. This is particularly evident in pediatric sarcomas such as rhabdomyosarcoma (RMS), which continue to represent a major therapeutic challenge. In addition, conventional treatments, including intensive chemotherapy, radiotherapy, and surgery, are associated with substantial long-term toxicities and functional impairment, underscoring the need for novel therapeutic strategies with reduced treatment burden.^1^ Thus, novel therapeutic strategies are urgently needed.

Immunotherapy holds particular promise for pediatric cancers, as it may avoid the genotoxicity of chemotherapy and irradiation as well as the functional disability caused by surgery, thereby reducing long-term complications.^1^ Tumor-infiltrating lymphocyte (TIL) therapy,^2^ T cell receptor (TCR)-engineered T cells such as afamitresgene autoleucel,^3^ and chimeric antigen receptor (CAR) T cell therapies^4^^,^^5^^,^^6^ are being actively investigated in sarcomas, with emerging clinical evidence demonstrating objective responses and durable disease control in a subset of patients. These advances highlight the potential of engineered immune cell therapies as a promising treatment modality for sarcomas. However, the efficacy of immunotherapy in sarcomas remains limited compared to hematological malignancies. Sarcomas are often characterized by low mutational burden and limited neoantigen expression and are therefore considered immunologically “cold tumors” with insufficient endogenous immune activation. In addition, the tumor microenvironment (TME) frequently lacks adequate co-stimulatory signals and is enriched with immunosuppressive components, which together limit the activation, persistence, and antitumor efficacy of adoptively transferred T cells, including CAR T cells. Among immunotherapeutic approaches, CAR T cell therapy has revolutionized the treatment of hematological malignancies but its efficacy in solid tumors has been modest.^7^ Two key barriers are the low expression of target antigens (TAs) and the immunosuppressive TME.^8^^,^^9^^,^^10^^,^^11^

We recently developed a ligand-based CAR T cell targeting Ephrin type-B receptor 4 (EPHB4),^12^ engineered via *piggyBac*-mediated gene modification. These EPHB4 CAR T cells exhibited potent antitumor activity in a RMS xenograft model and displayed a less exhausted T cell phenotype. However, further optimization is likely required for clinical efficacy.

Several groups have reported that lipid nanoparticle (LNP)-based CAR T boost vaccines, which promote TA expression in host antigen-presenting cells (APCs), enhance CAR T activity. Subcutaneous (s.c.) or intramuscular (i.m.) injection of such vaccines induces TA overexpression in APCs, significantly improving CAR T efficacy.^13^^,^^14^^,^^15^ LNPs have also been studied as non-viral delivery vehicles for direct tumor modification.^16^^,^^17^ Our group previously developed a novel delivery system using LNPs optimized with artificial intelligence to target specific cells.^17^ These LNPs efficiently deliver mRNA to tumor cells and have shown potent cytotoxicity when encapsulating inducible caspase-9 mRNA.^18^^,^^19^ To date, however, no studies have directly modulated the TME using an LNP-based vaccine to enhance CAR T function.

Co-stimulatory molecules (CSMs) are essential for sustaining and enhancing T cell function, including that of CAR T cells. Indeed, all clinically approved CAR T products incorporate 4-1BB or CD28 co-stimulatory domains to improve efficacy.^20^ Our group has also applied CSMs during CAR T production, using genetically modified feeder cells overexpressing both TA and CSMs, which promoted robust *ex vivo* CAR T expansion.^21^ This approach preserved CAR T stemness, underscoring the importance of physiological antigen presentation and co-stimulation in maintaining long-term memory function.

Based on these insights, we hypothesized that an LNP-based CAR T vaccine (CART-Vac) capable of inducing TA and CSM expression directly in tumor cells could enhance CAR T efficacy in solid tumors. In this study, we demonstrate that stable overexpression of both TA and CSM in genetically engineered RMS cells (Rh30) significantly augments the antitumor activity of EPHB4 CAR T cells *in vitro* and *in vivo*. Importantly, both components were required for optimal CAR T function. To facilitate clinical translation, we developed CAR-T vaccination (CART-Vac), a tumor-tropic LNP-based vaccine designed to induce TA and CSM expression in tumor cells, thereby markedly improving the antitumor effects of EPHB4 CAR T cells.

## Results

### Preparation of cell lines with artificial overexpression of TA and CSMs

**Figure 1:**
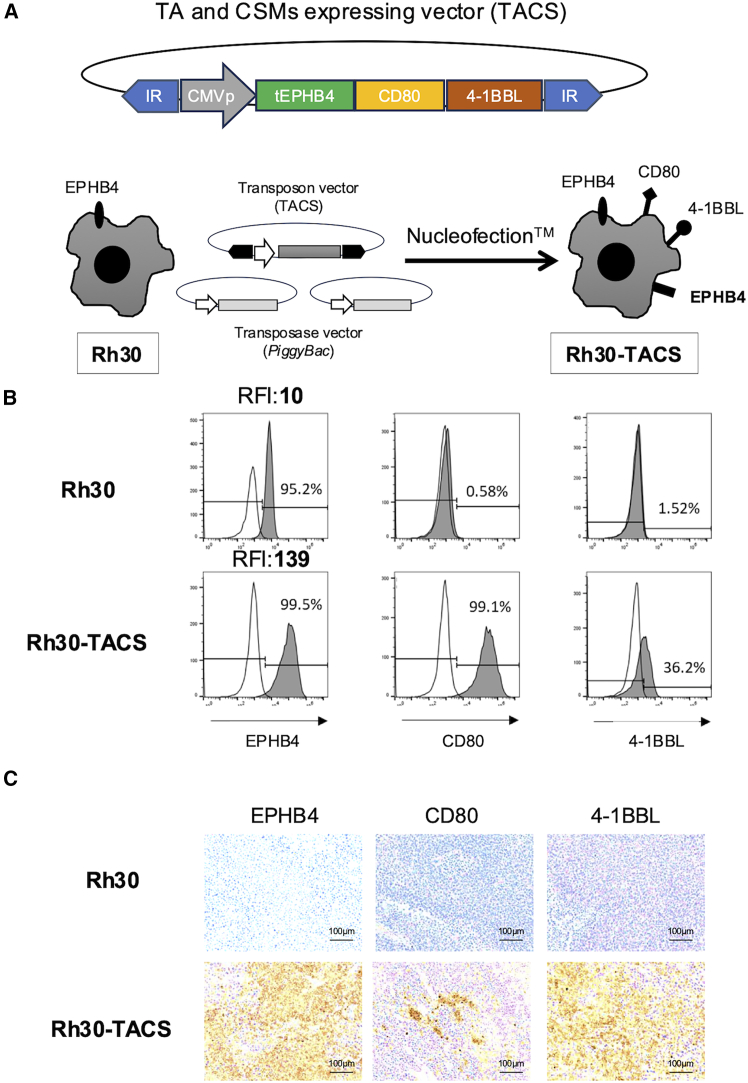
Establishment of tumors artificially overexpressing TA and CSMs (A) Schematic representation of the pIRII-tEPHB4-CD80-CD137L plasmid construct and preparation of Rh30-tEPHB4-80BBL by *piggyBac* gene modification. (B) EPHB4, CD80, and 4-1BBL expression levels in Rh30 and Rh30-TACS cells by flow cytometry. (C) Expression levels of EPHB4, CD80, and 4-1BBL in Rh30 (EPHB4^+^CD80-41BBL^−^) and Rh30-TACS (EPHB4^bright^ CD80 ^+^ 41BBL^+^) tumors in mice, assessed by IHC.

To generate tumor cells stably overexpressing TAs and CSMs, Rh30 cells were transfected with truncated EPHB4 (tEPHB4)—containing the extracellular and transmembrane domains plus a 20-amino acid cytoplasmic sequence—along with CD80 and 4-1BBL plasmids using *piggyBac* gene modification^22^ (Figure 1A). The cells were further purified using flow cytometry with an anti-4-1BBL antibody to establish Rh30 cells stably expressing TA and CSMs (Rh30-TACS). Flow cytometry confirmed CD80 and 4-1BBL expression in Rh30-TACS, whereas parental Rh30 cells were negative for both molecules (Figures 1B and S1). Although 95% of Rh30 cells originally expressed EPHB4, expression levels markedly increased after transduction, with relative fluorescence intensity rising from 10 to 139.

To assess stability, 2.0 × 10^6^ Rh30-TACS cells were s.c. injected into NOD.Cg-*Prkdc^scid^ Il2rg^tm1Wjl^*/SzJ (NSG) mice. On day 36, tumors were harvested and analyzed by immunohistochemical staining (Figure 1C). Tumors derived from Rh30-TACS were positive for EPHB4, CD80, and 4-1BBL, whereas those from wild-type Rh30 were weakly positive for EPHB4 but negative for CD80 and 4-1BBL. These findings confirmed that Rh30-TACS can be engrafted in immunocompromised mice with stable TA and CSM expression.

### Overexpression of TA and CSMs in tumor cells enhanced CAR T cell function

**Figure 2:**
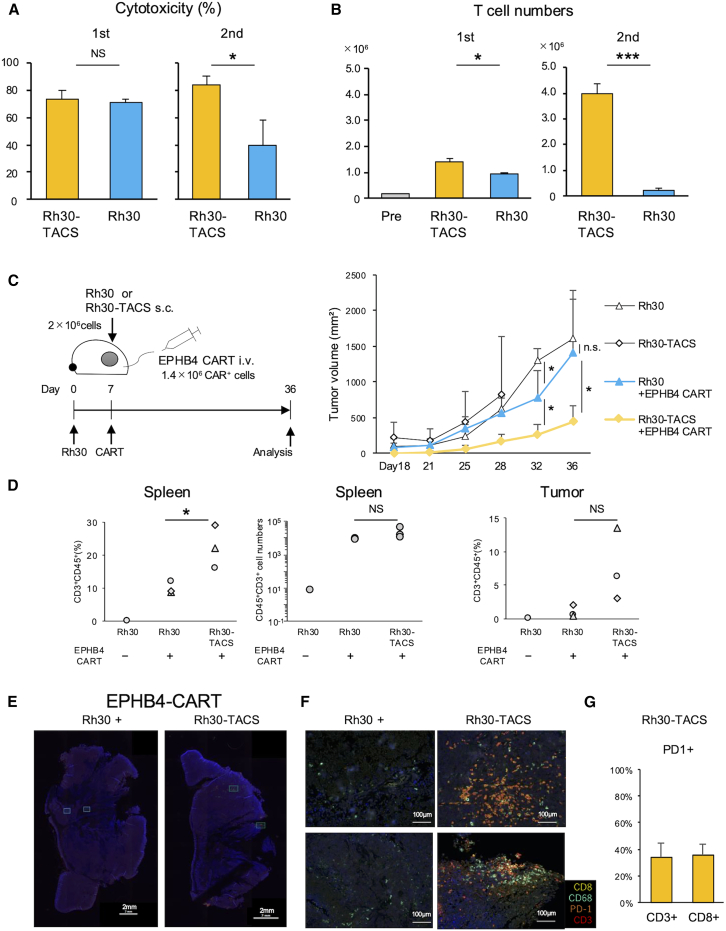
Overexpression of TA and CSMs enhances CAR T function *in vitro* and *in vivo* (A and B) EPHB4 CAR T cells were sequentially co-cultured with Rh30 (EPHB4^+^CD80-41BBL^−^), or Rh30-TACS (EPHB4^bright^ CD80 ^+^ 41BBL^+^). Tumor cells and T cells were quantified by flow cytometry, and cytotoxicity were calculated. Data represent mean values (*n* = 3). (C–G) *In vivo* antitumor effects of EPHB4 CAR T cells in Rh30- or Rh30-TACS-bearing mice. (C) Experimental design and sequential tumor volume evaluation. NSG mice were inoculated with 2.0 × 10^6^ Rh30 or Rh30-TACS cells. Seven days later, mice were treated with or without 1.4 × 10^6^ EPHB4 CAR T cells. Tumor volumes were compared among groups (mean ± *SD*, *n* = 3). (D) Numbers and percentages of CD45^+^CD3^+^ cells in the spleen and tumor, assessed by flow cytometry (*n* = 3). (E, F) Representative multiplex immunofluorescence images of Rh30- and Rh30-TACS-derived tumors treated with EPHB4 CAR T cells. Whole-slide images are shown in (E), and corresponding ×400 magnification views highlighting regions of immune cell infiltration are shown in (F). Primary antibodies included anti-CD3 (red), anti-CD8 (yellow), anti-PD-1 (orange), and anti-CD68 (green). Nuclei were counterstained with 4',6-diamidino-2-phenylindole (DAPI, blue). (G) Summary of the percentages of CD8^+^ and PD-1^+^ cells within CD3^+^ T cells in Rh30-TACS tumors. Data represent the mean ± SD of two sections per mouse from three mice. Statistical analyses: (A, B, F, and G) Student’s *t* test; ^∗^*p* < 0.05, ^∗∗^*p* < 0.01, ^∗∗∗^*p* < 0.001; i.v., (C and D) one-way ANOVA with Tukey’s multiple comparisons test. i.v., intravenous injection; s.c., subcutaneous (s.c.) injection.

To investigate whether tumor cells expressing TA and CSMs would enhance CAR T cell functions, EPHB4 CAR T cells were serially co-cultured with either Rh30 or Rh30-TACS cells. While EPHB4 CAR T cells showed comparable antitumor effects with both Rh30 and Rh30-TACS at first round (Figure 2A), CAR T cells exposed to Rh30-TACS exhibited significantly greater proliferation (Figure 2B) than those exposed to Rh30. Moreover, in the second round of co-culture, Rh30-TACS-primed EPHB4 CAR T cells demonstrated significantly enhanced cytotoxicity and T cell expansion compared with Rh30-primed CAR T cells (Figure 2A). Additionally, improved dose-dependent cytotoxicity was observed, accompanied by significantly increased T cell expansion in the second round of co-culture (Figure S2). These results suggest that overexpression of TA and CSMs in tumor cells enhances CAR T cell function *in vitro*.

To evaluate whether these findings translate *in vivo*, 2.0 × 10^6^ Rh30 or Rh30-TACS cells were inoculated into NSG mice, followed by intravenous (i.v.) administration of 1.4 × 10^6^ EPHB4 CAR T cells 7 days later (Figure 2C). Both Rh30 and Rh30-TACS tumors initially showed comparable growth (Figure 2C). In Rh30-inoculated mice, EPHB4 CAR T cells significantly suppressed tumor growth by day 32; however, the specific antitumor effects diminished after day 36, with tumor volume becoming similar to that in the tumor-only group. In contrast, EPHB4 CAR T cells significantly suppressed tumor growth in Rh30-TACS xenografts compared with Rh30 xenografts (Figure 2C). Furthermore, a higher percentage of CD45^+^CD3^+^ cells was observed in the spleens of Rh30-TACS xenografts than in Rh30 xenografts treated with EPHB4 CAR T cells (22.2% ± 6.5% vs. 9.7% ± 1.9%, *p* = 0.032), as detected by flow cytometry on day 36. Similarly, a trend toward increased CD45^+^CD3^+^ cell numbers in the spleen was observed in mice bearing Rh30-TACS compared with those bearing Rh30 (Figure 2D). Multiplex immunofluorescence (IF) imaging of tumor tissues revealed substantial infiltration of CD3^+^ and CD8^+^ T cells in two of three Rh30-TACS xenografts treated with EPHB4 CAR T cells (Figures 2E, 2F, S3, and S4). Approximately 35% of infiltrated CD3^+^ and CD8^+^ cells were positive for PD-1 in Rh30-TACS tumors treated by EPHB4 CAR T cells (Figure 2G). In contrast, this analysis could not be performed in control Rh30 tumors due to the limited number of tumor-infiltrating T cells. Collectively, these findings indicate that artificial overexpression of TA and CSMs in tumor cells promote CAR T cell expansion and enhances tumor control *in vivo*.

### TA expression is required for CSM-mediated enhancement of CAR T cell function *in vitro*

**Figure 3:**
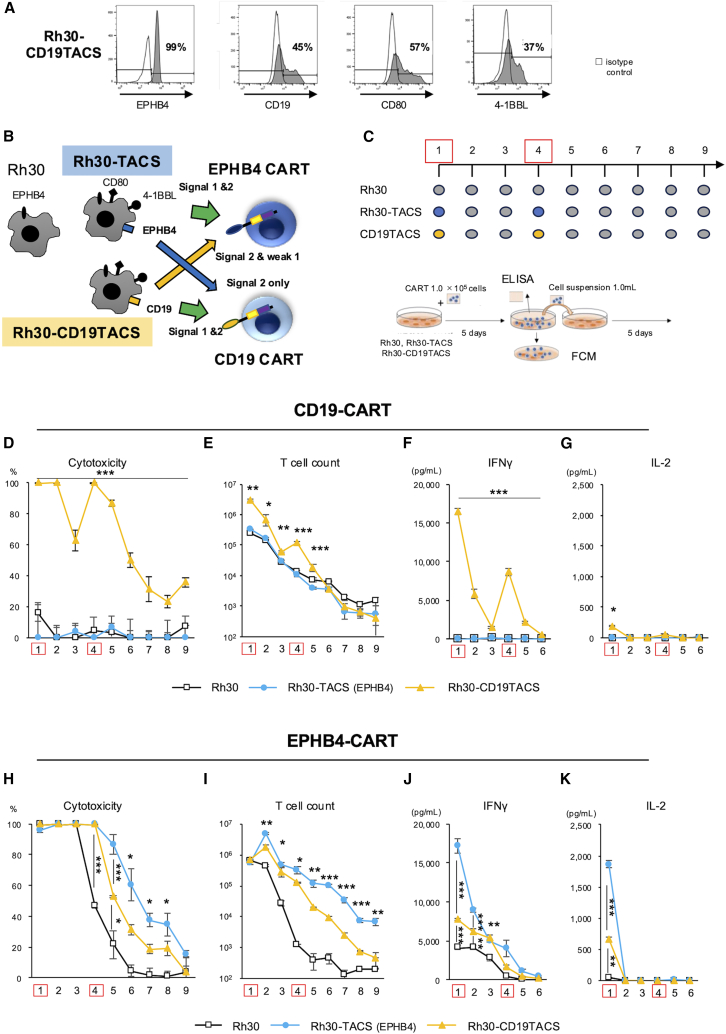
TA expression is required for CSM-mediated enhancement of CAR T cell function *in vitro* (A) Expression of EPHB4, CD19, CD80, and 4-1BBL in Rh30-CD19TACS cells, assessed by flow cytometry. (B) Hypothetical model of how TA- and CSM-expressing tumors (Rh30-TACS or Rh30-CD19TACS) affect CAR T cells. (C) Experimental design of sequential co-culture with Rh30, Rh30-TACS, or Rh30-CD19TACS and CD19 CAR T or EPHB4 CAR T cells. (D−K) Cytotoxicity, T cell counts, and cytokine secretion (IFN-γ or IL-2) of CD19 CAR T (D−G) or EPHB4 CAR T (H−K) cells against Rh30, Rh30-TACS, and Rh30-CD19TACS after sequential co-culture. (D−G) Cytotoxicity (D), T cell counts (E), and cytokine secretion (IFN-γ or IL-2). (F, G) of CD19 CAR T are shown. (H−K) Cytotoxicity (H), T cell counts (I), and cytokine secretion (IFN-γ or IL-2) (J, K) of EPHB4 CAR T are shown. Tumor and T cells were quantified by flow cytometry, and cytotoxicity and T cell expansion were calculated. (D, E, H, and I) Supernatants collected from co-cultures were analyzed for IFN-γ (pg/mL) (F, J) and IL-2 (pg/mL) (G, K) by ELISA. Data represent mean ± *SD* (*n* = 3).

We next investigated whether CSMs alone could enhance CAR T cell function. To this end, we generated Rh30 cells expressing another TA (CD19) together with CSMs (Rh30-CD19TACS; Figure 3A) and serially co-cultured them with either EPHB4 CAR T or CD19 CAR T cells (Figures 3B and 3C). Rh30-CD19TACS cells overexpressed CD19 and showed increased CD80 and 4-1BBL expression relative to parental Rh30 cells. EPHB4 expression was comparable between Rh30-CD19TACS and Rh30 cells but lower than in Rh30-TACS cells (Figure S1). Due to technical limitations, the proportions of TA^+^ and CD80^+^ cells were modestly lower in Rh30-CD19TACS than in Rh30-TACS cells (Figure S1).

As expected, CD19 CAR T cells showed minimal cytotoxicity and cytokine secretion against Rh30, which lacks TA and CSMs (Figure 3D). Similarly, CD19 CAR T cells displayed poor function against Rh30-TACS, which express CSMs but no TA. In contrast, CD19 CAR T cells demonstrated significantly enhanced cytotoxicity and IFN-γ secretion against Rh30-CD19TACS throughout multiple rounds of co-culture, with improved T cell persistence (Figure 3D). These results suggest that TAs are critical for adequate CAR T cell stimulation. Notably, once CD19 CAR T cells were activated through TA and CSMs by Rh30-CD19TACS in the first and fourth rounds of co-culture, they exhibited cytotoxicity and IFN-γ secretion against wild-type Rh30, which lacks TA and CSMs, during the second, third, and fifth to eighth rounds of co-culture (Figure 3D). This indicates potential antigen spreading induced by TA and CSM overexpression in the TME. CD19 CAR T cells secreted low levels of IL-2 only after the first round of co-culture against Rh30-CD19TACS.

We next asked whether overexpression of CSMs could enhance CAR T function in the presence of endogenous TA expression (Figures 3B and 3C). EPHB4 CAR T cells were serially co-cultured with Rh30, Rh30-CD19TACS, or Rh30-TACS. Although EPHB4 CAR T cells exhibited cytotoxicity and IFN-γ secretion against Rh30 by the fourth round of co-culture, likely due to weak TA expression, their activity diminished in later rounds (Figure 3E). In contrast, targeting Rh30-CD19TACS (expressing CSMs with baseline TA) significantly improved EPHB4 CAR T cytotoxicity, persistence, and IFN-γ secretion by the third round compared with Rh30 (Figure 3E). Furthermore, targeting Rh30-TACS (expressing both CSMs and enhanced TA) further prolonged cytotoxicity, persistence, and IFN-γ secretion compared with both Rh30 and Rh30-CD19TACS (Figure 3E). Importantly, once activated by Rh30-TACS, CAR T cells exhibited cytotoxicity, persistence, and IFN-γ secretion against wild-type Rh30 across multiple subsequent rounds (Figure 3E). These results suggest that although CSM overexpression enhances CAR T function in the presence of TA, TA overexpression may provide an additional improvement. However, because TA and CD80 expression levels were modestly lower in Rh30-CD19TACS than in Rh30-TACS due to technical limitations, reduced CSM expression may have contributed to the reduced activity of EPHB4 CAR T cells against Rh30-CD19TACS. EPHB4 CAR T cells secreted higher IL-2 levels against Rh30-TACS compared with Rh30 and Rh30-CD19TACS but only during the first round of co-culture. Thus, TA and CSM overexpression alone may not be sufficient to sustain IL-2 secretion by CAR T cells.

**Figure 4:**
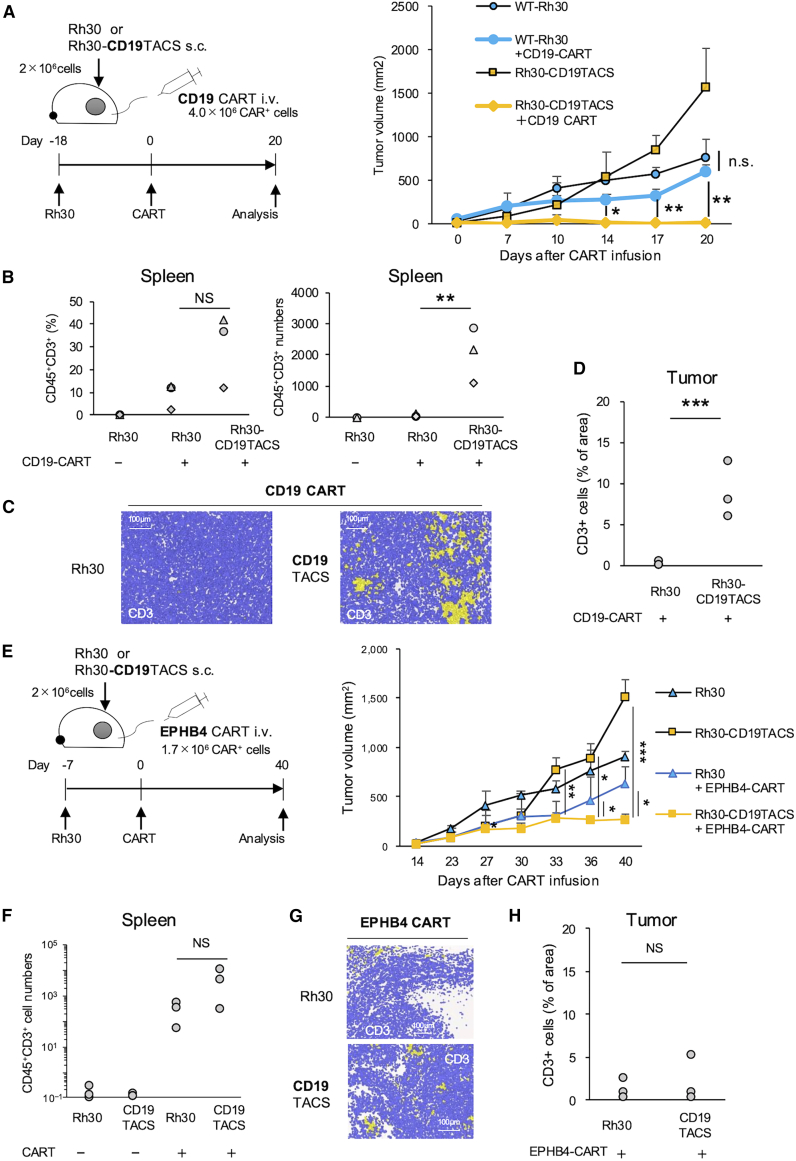
CSMs enhance CAR T activity only in the presence of innate TA expression *in vivo* (A–D) Effects of TA and CSM overexpression in tumors on CAR T function in a xenograft model. (A) Experimental design and sequential tumor volume evaluation. NSG mice were inoculated with 2.0 × 10^6^ Rh30 or Rh30-CD19TACS cells. Eighteen days later, mice were treated with or without 4.0 × 10^6^ CD19 CAR T cells. Tumor volumes were compared among groups (mean ± *SD*, *n* = 3). (B) Percentages and numbers of CD45^+^CD3^+^ cells in the spleen, assessed by flow cytometry (mean ± *SD*, *n* = 3). (C) Representative CD3^+^ infiltration in tumors by IHC. (D) Quantification of CD3^+^ infiltration (% area) in tumor tissue (two images per mouse, *n* = 3). (E–H) Effects of CSM overexpression with innate TA expression *in vivo*. (F) Experimental design and sequential tumor volume evaluation. Rh30 or Rh30-CD19TACS xenograft mice were treated with or without 1.7 × 10^6^ CD19 CAR T cells (mean ± *SD*, *n* = 3). (F) CD3^+^ cell counts in spleen by flow cytometry (mean ± *SD*, *n* = 3). (G) Representative CD3^+^ infiltration in tumors by IHC. (H) Quantification of CD3^+^ infiltration by IHC (two images per mouse, *n* = 3). Statistical analyses: one-way ANOVA with Tukey’s multiple comparisons test (A, B, E, F) and Students’ *t* test (D, H) were applied. i.v., intravenous injection; s.c., subcutaneous injection. ^∗^*p* < 0.05, ^∗∗^*p* < 0.01, ^∗∗∗^*p* < 0.001.

To validate these findings *in vivo*, we next treated xenografts of either Rh30 or Rh30-CD19TACS with CD19 CAR T cells. A total of 2.0 × 10^6^ Rh30 or Rh30-CD19TACS cells were s.c. injected into NSG mice (Figure 4A). Eighteen days post-inoculation, mice received i.v. injection of 4.0 × 10^6^ CD19 CAR T cells. CD19 CAR T cells completely inhibited Rh30-CD19TACS tumors, with tumor volume significantly suppressed compared with untreated controls (day 20, 19.0 ± 32.9 vs. 1,573.5 ± 625.8 mm^3^, *p* = 0.018; Figure 4A). Although wild-type Rh30 tumors grew more slowly than Rh30-CD19TACS, CD19 CAR T cells achieved significantly better control of Rh30-CD19TACS compared with wild-type Rh30 (day 20, 19.0 ± 32.9 vs. 613.6 ± 81.3 mm^3^, *p* = 0.003; Figure 4A). Moreover, CD45^+^CD3^+^ cell numbers were significantly higher in the spleens of Rh30-CD19TACS-bearing mice than controls 20 days after CAR T infusion (2,067 ± 888 vs. 64 ± 61, *p* = 0.018; Figure 4B). Immunohistochemistry (IHC) analysis also showed significantly higher percentages of tumor-infiltrating CD3^+^ cells in Rh30-CD19TACS-bearing mice (8.9 ± 3.5) compared with controls (0.23 ± 0.29, *p* = 0.012; Figures 4C and 4D). These results demonstrate that TA and CSM overexpression enhances CAR T cell activity *in vivo*.

Finally, to assess whether CSM overexpression alone could enhance CAR T function, we treated Rh30 or Rh30-CD19TACS xenografts with EPHB4 CAR T cells (Figure 4E). In this setting, Rh30-CD19TACS tumors expressed enhanced CSMs and baseline levels of TA. EPHB4 CAR T cells significantly suppressed tumor growth in Rh30-CD19TACS xenografts compared with Rh30-CD19TACS-only controls (day 40, 273.7 ± 61.7 vs. 1,513.2 ± 213.7 mm^3^, *p* < 0.001; Figure 4E). Although tumor growth was slower in Rh30 xenografts than Rh30-CD19TACS xenografts (day 40, 907.2 ± 63.7, *p* < 0.001), EPHB4 CAR T cells still achieved significantly better control against Rh30-CD19TACS compared with Rh30 (633.6 ± 211.6 mm^3^, *p* = 0.046; Figure 4E). A trend toward greater T cell expansion (Figure 4F) and increased tumor infiltration (Figures 4G and 4H) was observed, although not statistically significant. These results suggest that CSM overexpression alone can improve CAR T function in the presence of endogenous TA *in vivo*, consistent with *in vitro* observations.

### Development of an mRNA-LNP-based CAR T boost vaccine for overexpression of tumor-associated antigen and CSMs

**Figure 5:**
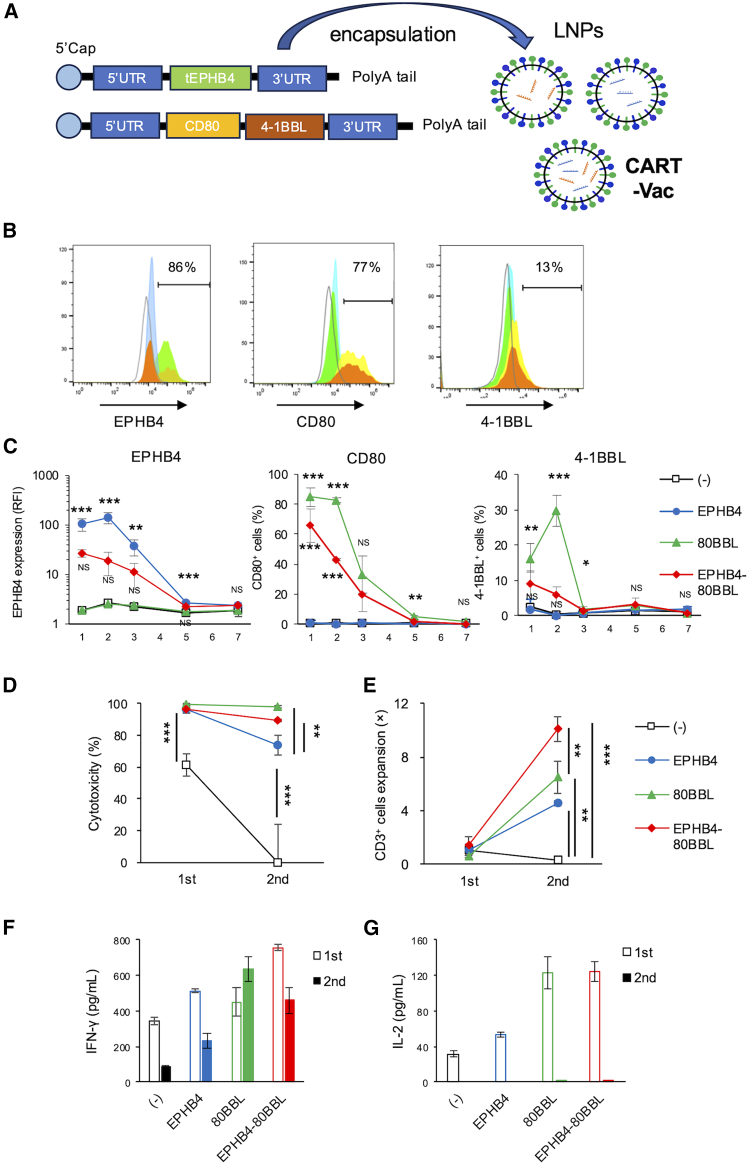
Development of tumor-tropic LNP-based CART-Vac to overexpress TA and CSMs in tumor cells (A) Schematic representation of tumor-tropic LNP-based CART-Vac. mRNAs encoding tEPHB4 or CD80 and 4-1BBL were generated and encapsulated individually or together in tumor-tropic LNPs. LNPs encapsulating both tEPHB4 and CD80-41BBL mRNAs were termed CART-Vac. (B) EPHB4, CD80, and 4-1BBL expression in Rh30 cells 24 h after LNP treatment, assessed by flow cytometry. Treatment groups are indicated by colors: light blue, no treatment; green, EPHB4; orange, CD80-41BBL; yellow, EPHB4 + CD80-41BBL. (C) Sequential analysis of EPHB4, CD80, and 4-1BBL expression levels in Rh30 cells by flow cytometry. Relative fluorescence intensity of EPHB4 expression to isotype control and % of CD80^+^ and 4-1BBL^+^ cells in Rh30 cells are shown. Data represent mean ± *SD* (*n* = 3). (D–G) Enhanced *in vitro* efficacy of CART-Vac with EPHB4 CAR T cells. Rh30 cells were treated with or without CART-Vac and subsequently with EPHB4 CAR T cells 24 h later. Cytotoxicity (D), CAR T expansion (E), IFN-γ secretion (F), and IL-2 secretion (G) are shown. Data represent mean ± *SD* (*n* = 3). Tumor and T cells were quantified by flow cytometry, and cytotoxicity and expansion were calculated. Supernatants were collected for IFN-γ and IL-2 measurement by ELISA. Statistical analyses: one-way ANOVA with Tukey’s multiple comparisons test (C−E) was applied.

We hypothesized that overexpression of TA and CSMs using an LNP-based CART-Vac could enhance CAR T efficacy. To this end, we employed tumor-tropic LNPs previously developed by our group,^18^^,^^19^ encapsulating mRNAs encoding TA and CSMs. mRNAs encoding truncated EPHB4 (tEPHB4), CD80, and 4-1BBL were generated and encapsulated in the LNPs (Figure 5A). LNPs encapsulating mRNAs for both tEPHB4 and CD80/4-1BBL were defined as CART-Vac.

To assess translation efficiency, Rh30 cells were treated with these LNPs. Target proteins were successfully expressed, with notably increased levels of EPHB4, CD80, and 4-1BBL in Rh30 cells at 24 h post-treatment (Figure 5B). Sequential analysis revealed that EPHB4, CD80, and 4-1BBL expression peaked at 24–48 h and gradually declined, reaching minimal levels by day 7 (Figure 5C). LNPs encoding EPHB4 significantly increased EPHB4 expression by day 5, while LNPs encoding CD80 and 4-1BBL elevated the percentages of CD80^+^ and 4-1BBL^+^ cells by day 2. CART-Vac (LNPs encoding EPHB4, CD80, and 4-1BBL) significantly enhanced the percentage of CD80^+^ cells in Rh30. CART-Vac also increased EPHB4 expression and the proportion of 4-1BBL^+^ cells, although these changes were not statistically significant (Figure 5C).

We next evaluated whether CART-Vac could enhance cytotoxicity *in vitro*. Based on prior experiments, Rh30 cells were treated with CART-Vac for 24 h and then serially co-cultured with EPHB4 CAR T cells. Overexpression of TA and CSMs by CART-Vac significantly enhanced CAR T cytotoxicity (Figure 5D) and proliferation (Figure 5E) compared with untreated controls in the second round of co-culture. CART-Vac also significantly increased IFN-γ and IL-2 secretion by CAR T cells (Figures 5F and 5G). These results suggest that CART-Vac-induced TA and CSM overexpression in tumor cells markedly enhances CAR T activity. Pretreatment with LNPs encoding either TA alone or CSMs alone also improved cytotoxicity and proliferation compared with untreated controls (Figures 5D and 5E). Notably, CSM-only overexpression induced greater cytotoxicity, proliferation, and cytokine secretion than TA-only overexpression (Figures 5D–5G), suggesting that CSMs play a more critical role than TA in enhancing CAR T activity in the presence of baseline TA expression.

**Figure 6:**
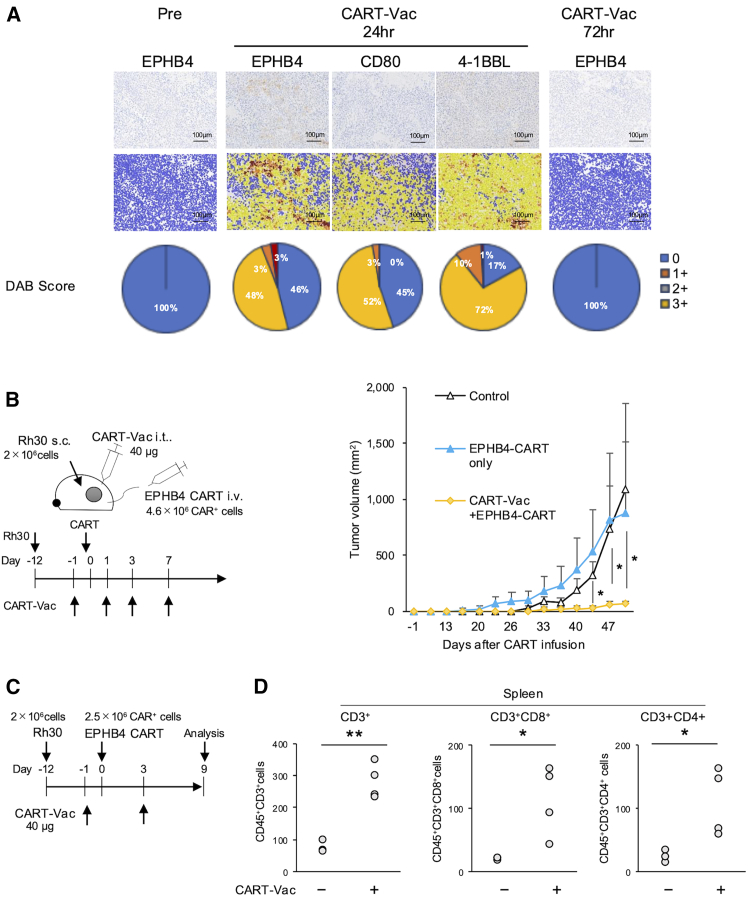
CART-Vac enhances antitumor effects and proliferation of EPHB4 CAR T cells *in vivo* (A) Validation of EPHB4, CD80, and 4-1BBL expression in tumors by IHC after intratumoral (i.t.) CART-Vac administration. Expression at baseline and at 24 and 72 h post-treatment is shown. DAB scores (%) were calculated as the proportion of DAB-positive cells among all cells (mean of two to six images per tissue). The IHC image at pretreatment (top) was reused from Figure 1C. (B) Experimental design and sequential tumor volume evaluation. Antitumor effects of combination therapy with EPHB4 CAR T cells with or without CART-Vac were assessed in Rh30-inoculated mice (mean ± *SD*, *n* = 3). (C) Experimental schematic. (D) Numbers of CD45^+^CD3^+^ cells in spleens, quantified by flow cytometry (*n* = 3–4). Statistical analyses: (B) One-way ANOVA with Tukey’s multiple comparisons test; (D) Student’s *t* test. i.v., intravenous injection; i.t., intratumoral injection; s.c., subcutaneous injection.

To validate transcription efficiency *in vivo*, NSG mice bearing Rh30 tumors received intratumoral (i.t.) CART-Vac injections. Tumors collected at 24 and 72 h post-treatment were analyzed by IHC. At 24 h, approximately 54% of tumor cells were positive for EPHB4, whereas minimal EPHB4 expression was detected at 72 h (Figure 6A). CD80 and 4-1BBL expression was also evident 24 h after CART-Vac treatment (Figure 6A).

Finally, we investigated the efficacy of combining CART-Vac with EPHB4 CAR T cells *in vivo*. NSG mice bearing Rh30 tumors received i.t. CART-Vac injection on day -1 (Figure 6B). After 24 h, mice were treated i.v. with 4.6 × 10^6^ EPHB4 CAR T cells, followed by a second CART-Vac injection 24 h post-CAR T infusion. While EPHB4 CAR T cells alone showed minimal antitumor effect, combination therapy significantly inhibited tumor growth (day 50, 68.8 ± 20.7 vs. 1,315.5 ± 183.6 mm^3^, *p* = 0.0011; Figure 6B).

To elucidate the mechanism underlying enhanced efficacy, spleens and tumors were collected on day 9 in a separate experiment (Figure 6C). Although CD3^+^ cells were scarcely detected in tumors of the combination group (data not shown), flow cytometry revealed significantly higher numbers of CD45^+^CD3^+^ human T cells in spleens of combination-treated mice compared with controls (76.0 ± 18.2 vs. 280.0 ± 54.9, *p* = 0.0017; Figure 6D). Similarly, CD45^+^CD8^+^ (20.0 ± 2.6 vs. 112.1 ± 55.7, *p* = 0.038) and CD45^+^CD4^+^ (25.4 ± 9.5 vs. 110.3 ± 53.4, *p* = 0.045) cells were also significantly increased in the combination group (Figure 6D).

To determine whether CSM overexpression alone is sufficient to enhance CAR T cell function, we performed a similar experiment using CART-Vac targeting an irrelevant antigen, CD19. Although the combination of CD19CART-Vac and EPHB4 CAR T cells showed trends toward improved survival, enhanced tumor control, and increased T cell proliferation in the spleen, these differences were not statistically significant (Figure S5). These results indicate that co-expression of both the TA and CSMs is necessary to achieve robust CART-Vac activity. Collectively, these findings suggest that CART-Vac significantly promotes CAR T cell expansion, resulting in superior tumor control *in vivo*.

## Discussion

We demonstrated that the overexpression of TA and CSMs significantly enhanced CAR T cell function both *in vitro* and *in vivo* using artificial tumor models, Rh30-TACS and Rh30-CD19TACS. Furthermore, by employing an mRNA-LNP-based CART-Vac to directly modify tumor cells to overexpress TA and CSMs, we successfully improved the antitumor efficacy of CAR T cells. These enhanced functions were evidenced by increased cytotoxicity, T cell proliferation, and elevated levels of Th1 cytokines. While this study focuses on a pediatric sarcoma model, the underlying concept of augmenting antigen presentation and co-stimulation within the TME is not restricted to pediatric malignancies. Rather, this approach may be broadly applicable to a wide range of immunologically “cold” solid tumors. Moreover, as LNP-mRNA technologies continue to evolve rapidly,^23^^,^^24^ incorporation of more advanced and fine-tuned systems may further enhance the therapeutic potential of this strategy in the future.

Weak and heterogeneous expression of TAs remains a major challenge in CAR T therapy for solid tumors. Additionally, the hostile TME significantly impairs CAR T efficacy and persistence in patients with solid malignancies. Several efforts have been made to overcome these challenges, including the modification of CAR structures and combination therapies with other treatment modalities.^10^ Among them, booster vaccines represent a promising approach.^9^^,^^25^

Recent studies have investigated CAR T booster vaccines using *ex vivo* gene transfer of tumor antigens into dendritic cells (DCs) as well as mRNA-based gene transfer of CAR TAs into DCs and other APCs.^15^^,^^26^ Multiple groups have demonstrated the synergistic effects of CART-Vac by utilizing host APCs to stimulate CAR T cells through overexpression of TAs. However, to the best of our knowledge, no prior studies have employed tumor cells themselves as APCs to enhance CAR T function. Our study, therefore, proposes a novel treatment strategy in which CART-Vac utilizes tumor cells to augment CAR T efficacy by overexpressing TAs and CSMs.

Although i.t. injection of the vaccine may limit clinical application, the generally higher transduction efficiency in tumor cells compared with normal APCs suggests that this approach holds promise for directly modifying the TME. For tumor types such as RMS, which are typically accessible but unresectable, i.t. administration of CART-Vac could be a feasible strategy to enhance CAR T function. Further studies are warranted to optimize this approach, particularly for solid tumors accessible via the body surface.

Our mechanistic investigations revealed that both TA and CSM expression in the TME were critical for enhancing CAR T function. Overexpression of CSMs alone did not enhance CD19 CAR T activity in tumor models lacking endogenous CD19 expression. In contrast, co-expression of CSMs and CD19 (as an irrelevant TA) enhanced the function of EPHB4 CAR T cells. Although we could not fully rule out the possibility that the reduced CAR T activity against Rh30-CD19TACS was due to lower TA and CD80 expression compared with Rh30-TACS, these findings suggest that even when expressed even at low and heterogeneous levels, an endogenous TA is more effective than an irrelevant TA in enhancing CAR T function when delivered via a boost vaccine strategy.

CSMs provide the “signal 2” required for T cell activation. Incorporation of co-stimulatory domains in CAR constructs has significantly improved CAR T efficacy and contributed to clinical successes.^20^ The presence of CSM domains such as 4-1BB or CD28 in all approved CAR T products underscores their importance.^20^ Additionally, studies with armed oncolytic viruses have demonstrated that overexpression of CSMs such as CD80,^27^ CD40L, and 4-1BBL^28^ in the TME significantly enhances antitumor activity. Therefore, combining CSM expression with TA expression should represent a promising strategy to potentiate CART-Vac activity.

Consistent with recent reports on CAR T booster vaccines,^13^ our findings showed that overexpression of TA and CSMs in tumor cells significantly enhanced IFN-γ secretion by CAR T cells, resulting in improved cytotoxicity and differentiation toward a Th1 phenotype. Elevated IFN-γ is also known to upregulate MHC class I expression and modulate adhesion factor expression in tumor cells, which may further improve CAR T activity. Moreover, IFN-γ can influence neoangiogenesis and tumor stroma, while also recruiting and activating host immune cells, thereby enhancing tumor antigen-specific cytotoxic T lymphocyte (CTL)-mediated cytotoxicity.^9^^,^^29^^,^^30^^,^^31^ Notably, Rh30-CD19TACS-treated CD19 CAR T cells elicited potent cytotoxic activity against Rh30 cells lacking TA and CSM expression, indicating potential antigen spreading. Recent studies have shown that CART-Vac improves CAR T function through antigen spreading, potentially overcoming resistance via antigen escape.^13^^,^^32^ Although we did not directly confirm this, our data suggest that TA and CSM overexpression enhances IFN-γ secretion in the TME, which may drive antigen spreading and warrants further study.

Interestingly, we observed relatively weak IL-2 secretion by EPHB4 CAR T cells, even in the presence of TA and CSM overexpression in tumor cells. Because IL-2 plays a key role in T cell proliferation and persistence in CAR T therapy, additional modifications, such as induction of cytokine release using LNP-mRNA,^24^ may be required to augment IL-2 secretion and improve CAR T function.

In this study, TA and CSM overexpression induced sustained CAR T proliferation upon repeated tumor cell stimulation, despite increased PD-1 expression on CAR T cells. Prior work has shown that CD80 interacts with PD-L1 in cis on APCs, thereby preventing PD-L1/PD-1 binding and T cell inhibition.^33^ Thus, expression of CD80 on tumor cells may interact with PD-L1 and protect CAR T cells from exhaustion, supporting their sustained proliferation.

### Limitations

This study has several limitations. First, we only used xenograft models in immunocompromised NSG mice, which lack human immune components. More representative models, such as humanized NSG mice, are needed to recapitulate the TME and host immune responses. In addition, we were unable to evaluate the effects of CART-Vac on the *in vivo* cytokine profiles of CAR T cells, which would provide valuable mechanistic insight into the effects of CART-Vac *in vivo*. Second, this study did not assess in detail the CAR T phenotypes affected by CART-Vac. Advanced analyses, such as multiparameter flow cytometry or single-cell RNA sequencing, are required to clarify these effects. Finally, although our LNP-based CART-Vac is tumor-tropic and efficient at mRNA delivery, protein expression was transient, nearly disappearing within 72 h. Achieving longer and stronger protein expression, for example through the incorporation of circular RNA,^23^ would likely provide greater boosting effects and should be pursued in future studies.

### Conclusion

In conclusion, we demonstrated that overexpression of TA and CSMs in tumors significantly enhances CAR T function, improving cytotoxicity, proliferation, and cytokine secretion *in vitro* and *in vivo*. Moreover, the use of tumor-tropic, LNPs enabled CART-Vac to induce TA and CSM overexpression in tumor cells, resulting in significantly improved CAR T activity. Both TA and CSMs are required for CART-Vac-mediated CAR T enhancement. This combinatorial strategy holds promise as an alternative approach to improve the efficacy of CAR T therapy for solid tumors.

## Material and methods

### Ethical approval

This study was conducted in accordance with the Declaration of Helsinki and was approved by the Institutional Review Board of the School of Medicine, Shinshu University (approval no. 4855).

### Plasmids

The *PiggyBac* (PB) transposon plasmids used for the stable expression of the EPHB4 CAR (pIRII-EPHB4-CAR-28z)^7^ and the CD19-specific CAR transgene (pIRII-CAR.CD19-28z-CH2CH3-free)^34^ have been described previously. The pIRII-tr tEPHB4-CD80-CD137L plasmid contains sequences encoding the extracellular, transmembrane, and 20-amino acid intracellular regions of the EPHB4 protein, fused to full-length CD80 and 4-1BBL (CD137L) via a self-cleaving 2A sequence,^12^ and was used to establish Rh30-TACS cells. Similarly, the pIRII-tCD19-CD80-CD137L plasmid contains truncated CD19 sequences encoding the extracellular, transmembrane, and 20-amino acid intracellular regions of the CD19 protein, fused to full-length CD80 and 4-1BBL (CD137L) via a self-cleaving 2A sequence, as previously described,^12^ and was used to establish Rh30-CD19TACS cells.

### Cell lines and human blood samples

The EPHB4-positive sarcoma cell line Rh30 was obtained from the American Type Culture Collection (Manassas, VA, USA). To establish genetically modified Rh30 cells overexpressing EPHB4, CD80, and 4-1BBL (Rh30-TACS) or CD19, CD80, and 4-1BBL (Rh30-CD19TACS), 1 × 10^6^ Rh30 cells were transfected with 7.5 μg of either pIRII-tEPHB4-CD80-CD137L (for Rh30-TACS) or pIRII-tCD19-CD80-CD137L (for Rh30-CD19TACS), together with 7.5 μg of pCMV-PB. Transfections were performed using a 4D-Nucleofector device (program FL-115) and the P3 Primary Cell 4D-Nucleofector X Kit (Lonza). Transfected cells were purified by flow cytometry with a BD FACSAria III cell sorter (BD Biosciences) and maintained in Dulbecco’s modified Eagle’s medium (Thermo Fisher Scientific) supplemented with 10% fetal bovine serum (Serana Europe GmbH) and 1% penicillin-streptomycin (Gibco) at 37°C in a humidified incubator with 5% CO_2_.

Peripheral blood samples were collected from healthy volunteers after obtaining written informed consent, in accordance with the Declaration of Helsinki. Peripheral blood mononuclear cells (PBMCs) were isolated by density gradient centrifugation using Lymphocyte Separation Medium 1077 (FUJIFILM Wako Pure Chemical Corporation) for CAR T cell generation.

### Generation of PBMC-derived, *piggyBac* transposon-mediated CAR T cells

EPHB4 CAR T cells were generated using *piggyBac* modification, as described previously.^7^ Briefly, 4 × 10^7^ PBMCs were electroporated with pCMV-PB (7.5 μg) and either pIRII-EPHB4-CAR-28z (7.5 μg) or pIRII-CD19-CAR-28z (7.5 μg) using the MaxCyte ATX electroporator (MaxCyte) with protocol RTC 14-3. In parallel, 1 × 10^7^ PBMCs from the same donor were electroporated with either pIRII-tEPHB4-CD80-CD137L (15 μg) or pIRII-tCD19-CD80-CD137L (15 μg) to generate feeder cells. CAR T cells and feeder cells were cultured in complete culture medium (CCM), consisting of ALyS705 medium (Cell Science & Technology Institute) supplemented with 5% artificial serum (Cell Science & Technology Institute), 10-ng/mL IL-7, and 5-ng/mL IL-15 (Miltenyi Biotec).

About 24 h after electroporation, feeder cells were inactivated by ultraviolet irradiation and subsequently co-cultured with CAR T cells in CCM. Cells were harvested on day 14 and used for downstream experiments.

### Lipids

The functional lipid FFT-20 was synthesized as previously described.^18^ 1,2-dioleoyl-sn-glycero-3-phosphoethanolamine (DOPE), 1,2-dioleoyl-3-trimethylammonium-propane chloride salt (DOTAP), and cholesterol were purchased from Sigma-Aldrich. DMG-PEG2000 (1,2-dimyristoyl-rac-glycero-3-methylpolyoxyethylene) was obtained from Merck KGaA. Because of its optimal translation efficiency in Rh30 cells, LNP-43^19^ was used for all experiments.

### mRNA synthesis

mRNA was synthesized using standard *in vitro* transcription with the T7 mScript Standard mRNA Production System (CELLSCRIPT), according to the manufacturer’s instructions. Briefly, plasmid DNA encoding tEPHB4 or CD80-CD137L was linearized overnight and subsequently transcribed *in vitro* using the T7 mScript system to generate capped and polyadenylated mRNA.

### Preparation of LNPs

An mRNA solution (0.18 mg/mL) was prepared in 10 mM HEPES buffer (pH 7.3; Merck). A lipid mixture consisting of FFT-20, DOPE, DOTAP, cholesterol, and DMG-PEG2000 at a molar ratio of 25.8:4.9:9.8:55.8:3.7 was dissolved in ethanol and mixed with the mRNA under vortexing. The mixture was then diluted 10-fold with 10 mM HEPES (pH 7.3) and concentrated by ultrafiltration using Amicon Ultra 0.5 Ultracel-50 devices (50-kDa cutoff; Merck).

### Flow cytometry

Flow cytometry was performed using BD FACS Accuri or Lyric systems (BD Biosciences), following the manufacturer’s instructions, and analyzed with FlowJo software (Tree Star, Inc.). Detailed antibody information is provided in Table S1.

### *In vitro* killing assay

To evaluate cytotoxicity, EPHB4-CAR T cells were co-cultured with target cells for 5 days at an effector-to-target (E:T) ratio of 1:1. After co-culture, cells were harvested and stained with 50,000 CountBright Absolute Counting Beads (Invitrogen) and 7-amino-actinomycin D (7AAD). The numbers of viable tumor cells (GFP^+^, CD3^-^, and 7AAD^−^) and T cells (CD3^+^, GFP^−^, and 7AAD^−^) were quantified by flow cytometry.

For serial co-culture experiments, 1.0 × 10^5^ target cells and EPHB4 CAR T cells were seeded at the indicated E:T ratios in 24-well plates. On day 5, half of the cells were analyzed by flow cytometry, and 1.0 × 10^5^ fresh target cells were added to the remaining cultures.

### ELISA assay

Co-culture supernatants were collected 5 days after initiation and analyzed for cytokine production. IFN-γ and IL-2 concentrations were measured using human IFN-γ and IL-2 DuoSet ELISA kits (R&D Systems), according to the manufacturer’s instructions.

### Animal experiments

All animal procedures were approved by the Institutional Animal Care and Use Committee of Shinshu University School of Medicine. NOD.Cg-*Prkdc*^*scid*^*Il2rg*^*tm1Wjl*^*/SzJ* (NSG) mice were obtained from Charles River Laboratories (Wilmington, MA, USA). Tumor size was measured sequentially with calipers (Mitutoyo Corporation), and tumor volume was calculated. To evaluate CAR T cell infiltration *in vivo*, spleen and tumor samples were collected, and CAR T cell numbers were quantified by flow cytometry. T cell doses were adjusted based on CAR^+^ cell counts.

At the same time, formalin-fixed, paraffin-embedded tumor sections were prepared for quantitative multiplex IHC. All mice were euthanized according to predefined ethical criteria.

### IHC staining and analysis

IHC was performed on 4-μm-thick sections using the Opal Multiplex IHC Assay Kit (PerkinElmer). Antigen retrieval was carried out with either EDTA buffer (pH 9.0; Dako) or citrate buffer (pH 6.0; Abcam). Rodent Block (Dako) was applied to minimize background staining.

Primary antibodies included mouse monoclonal anti-EPHB4 (Santa Cruz Biotechnology), rabbit monoclonal anti-CD80 (Abcam), mouse monoclonal anti-4-1BBL (Abcam), and rabbit monoclonal anti-CD3 (Abcam). Horseradish peroxidase (HRP)-conjugated anti-mouse or anti-rabbit secondary antibodies (EnVision+ Single Reagents; Dako) were used for detection. Sections were incubated with 3,3′-diaminobenzidine (DAB) substrate solution (Dako), counterstained with Mayer’s hematoxylin, and mounted with GVA Mount (Zymed Laboratories).

Images were acquired with the Vectra 3 automated quantitative pathology imaging system (PerkinElmer) and analyzed using InForm software. For quantitative analysis, two to eight randomly selected regions of interest (ROIs) per section were evaluated. Detailed antibody information is provided in Table S2.

### Multiplex IF staining and analysis

Immunofluorescence (IF) staining was performed using the Opal 7-Color Manual IHC Kit (Akoya Biosciences) with in-house optimization. Primary antibodies included rabbit monoclonal anti-CD3, rabbit monoclonal anti-CD4, mouse monoclonal anti-CD8, rabbit monoclonal anti-PD-1 (1:250), rabbit monoclonal anti-EphrinB2 (1:100), and mouse monoclonal anti-CD68 (1:200; all from Abcam). HRP-conjugated anti-mouse/rabbit (HRP Ms + Rb; Akoya Biosciences) served as the secondary antibody.

Images were acquired using the Vectra 3 system (PerkinElmer) and analyzed with InForm software. For quantitative analysis, two ROIs were selected per section. Detailed antibody information is provided in Table S2.

### Statistical analysis

Statistical analyses were performed using EZR v.1.37 (Saitama Medical Center, Jichi Medical University).^35^ Comparisons between two groups were conducted using Student’s *t* test, whereas comparisons among three or more groups were performed using one-way analysis of variance (ANOVA). A *p* value < 0.05 was considered statistically significant.

## Data and code availability

The datasets generated and/or analyzed during the current study are available from the corresponding author on reasonable request.

## Acknowledgments

The authors thank Enago for providing an English-language review services.

This study was conducted using research equipment shared under the MEXT Project for promoting public utilization of advanced research infrastructure (program for supporting construction of core facilities; grant JPMXS0441000021). This study was also supported by the 10.13039/501100001691Japan Society for the Promotion of Science (10.13039/501100001691JSPS) 10.13039/501100001691KAKENHI (20K08227 and 23K07247) awarded to S.S. and by collaborative research funding from Toshiba Corporation provided to Y.N.

## Author contributions

Conceptualization, S.S., S.Y., and Y.N.; methodology, I.N., S.S., E.A., A.H., S.Y., M.S.-I., and Y.N.; investigation, I.N., S.S., J.Z., M.T., and E.A.; writing – original draft, I.N. and S.S.; writing – review and editing, S.S., S.Y., and Y.N.; funding acquisition, S.S. and Y.N.; resources, S.S., E.A., S.Y., M.S.-I., and A.H.; supervision, S.S., S.Y., and Y.N.

## Declaration of interests

This study was conducted collaboratively between Shinshu University and Toshiba Corporation. Y.N. received a research grant from Toshiba Corporation. E.A. and M.S.-I. are employees of Toshiba Corporation. Shinshu University and Toshiba Corporation have filed joint patent applications related to liposomal delivery of therapeutic genes, with S.S., E.A., M.S.-I., and Y.N. named as inventors. M.T. has stock ownership in A-SEEDS Co., Ltd. S.Y. is the Chief Executive Officer of A-SEEDS Co., Ltd., with equity, and Y.N. is an Executive Officer of A-SEEDS Co., Ltd., with equity. Y.N. also received research funding from BrightPath BioTherapeutics Co., Ltd., Bourbon Corp., and CSTI Inc.

## Declaration of generative AI and AI-assisted technologies in the writing process

During manuscript preparation, the authors used ChatGPT to improve language and readability. After using this tool, the authors carefully reviewed and edited the content and take full responsibility for all aspects of the publication.

## Supplemental information

Document S1. Figures S1–S5, Tables S1, and S2

Document S2. Article plus supplemental information
