# Supplementary material for: mRNA-LNP vaccine providing antigen and co-stimulation in the tumor microenvironment enhances CAR T cell function (CART-Vac)
Source: Mol Ther Oncol. 2026 May 14;34(2):201234. doi: 10.1016/j.omton.2026.201234 (PMC13233755; doi:10.1016/j.omton.2026.201234)
Supplement: Document S1. Figures S1–S5, Tables S1, and S2 [file mmc1.pdf]

OMTON, Volume *34*

Supplemental information

mRNA-LNP vaccine providing antigen and co-stimulation in the tumor microenvironment enhances CAR T cell function (CART-Vac)

Ikumi Nakashima, Shoji Saito, Jingbo Zhao, Miyuki Tanaka, Eiichi Akahoshi, Aiko Hasegawa, Mitsuko Sugano-Ishihara, Shigeki Yagyu, and Yozo Nakazawa

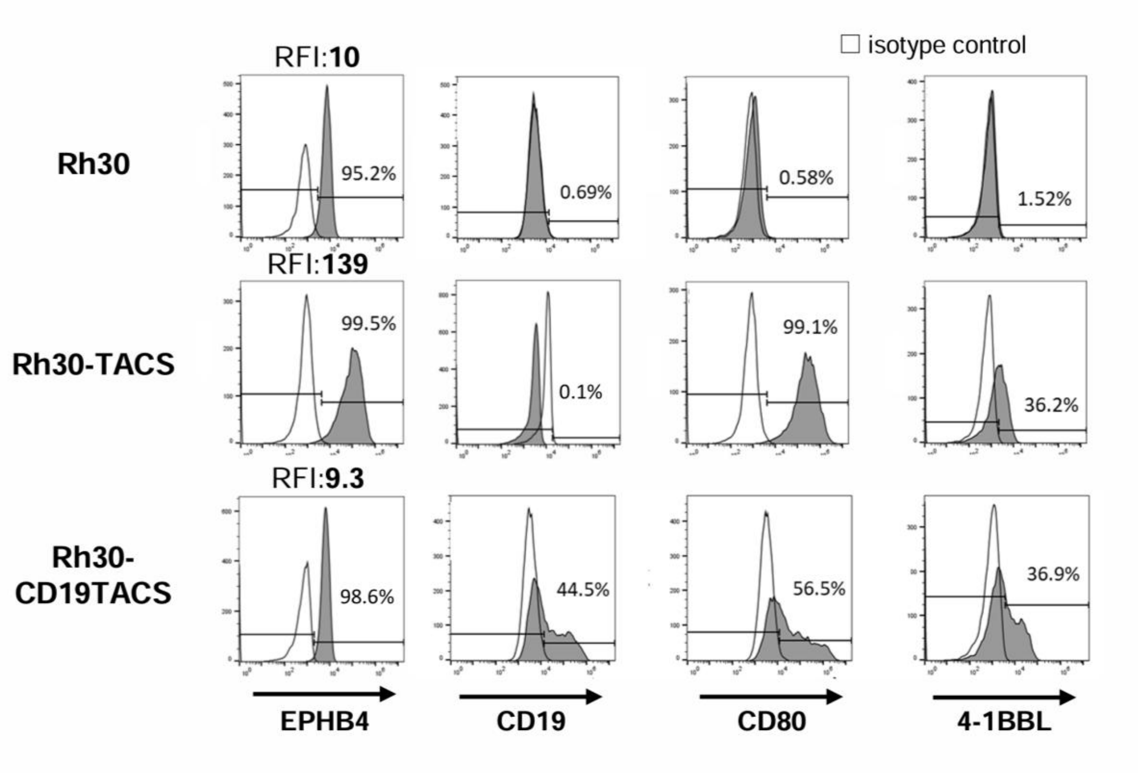

Figure S1. Summary of TA and CSM expression in artificially overexpressing Rh30 cells.

EPHB4, CD80, and 4-1BBL expression levels in Rh30, Rh30-TACS, and Rh30-CD19TACS cells by flow cytometry. The data were reused from Figure 1B and 3A.

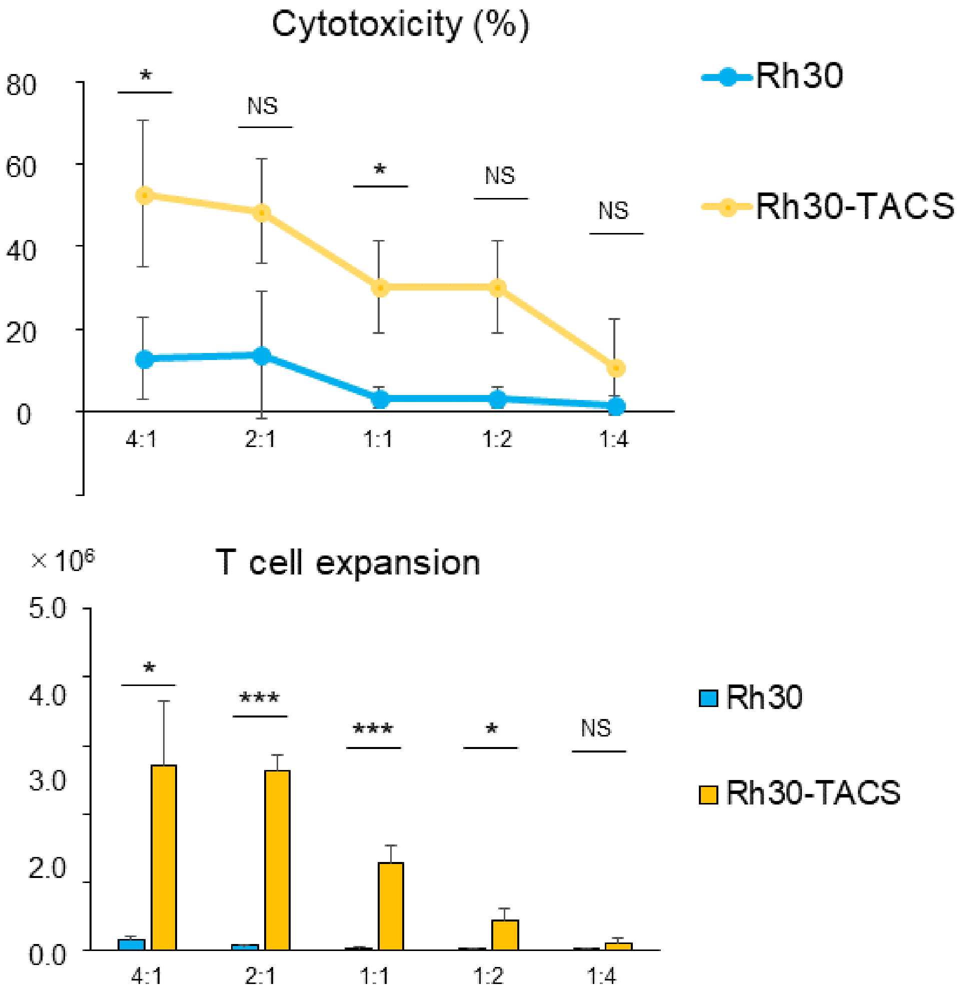

Figure S2. Enhanced dose-dependent cytotoxicity and T cell expansion of Rh30-TACS-treated EPHB4 CAR-T cells.

EPHB4 CAR-T cells co-cultured with Rh30 ($EPHB4^{+}CD80^{-}41BBL^{-}$) or Rh30-TACS ($EPHB4^{bright}CD80^{+}41BBL^{+}$) were rechallenged with Rh30 or Rh30-TACS cells at various effector : target (E:T) ratios. Six days later, tumor cells and T cells were quantified by flow cytometry, and cytotoxicity were calculated. Data represent mean ± SD (n = 3). Student's *t* test. $^{*}p < 0.05$. $^{**}p < 0.01$. $^{***}p < 0.001$.

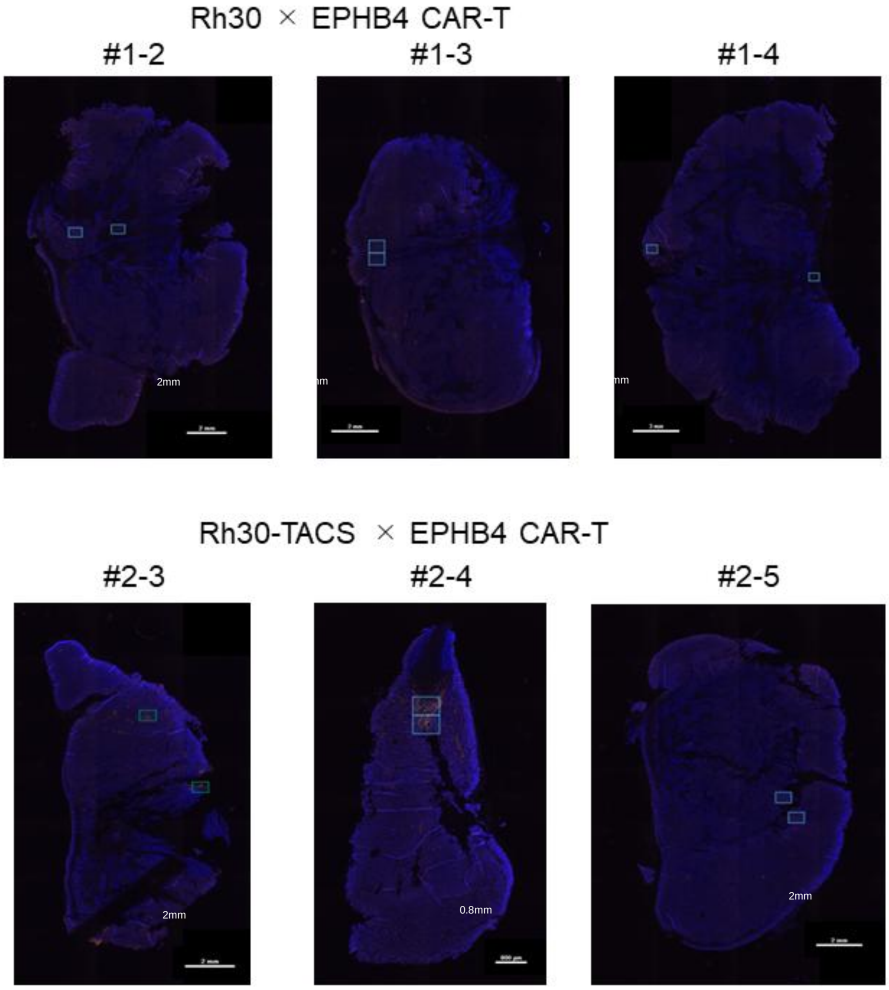

Figure S3. Whole-slide scan of immunohistochemical staining of the tumor
Multiplex immunofluorescence imaging and tumor analysis. Whole-slide images of tumors from Rh30- and Rh30-TACS–bearing mice treated with EPHB4 CAR-T cells (n = 3 per group). Primary antibodies included anti-CD3 (red), anti-CD8 (yellow), anti-PD-1 (orange), and anti-CD68 (green). Nuclei were counterstained with DAPI (blue). Images corresponding to mouse #1-2 and #2-3 were reused from Figure 2E.

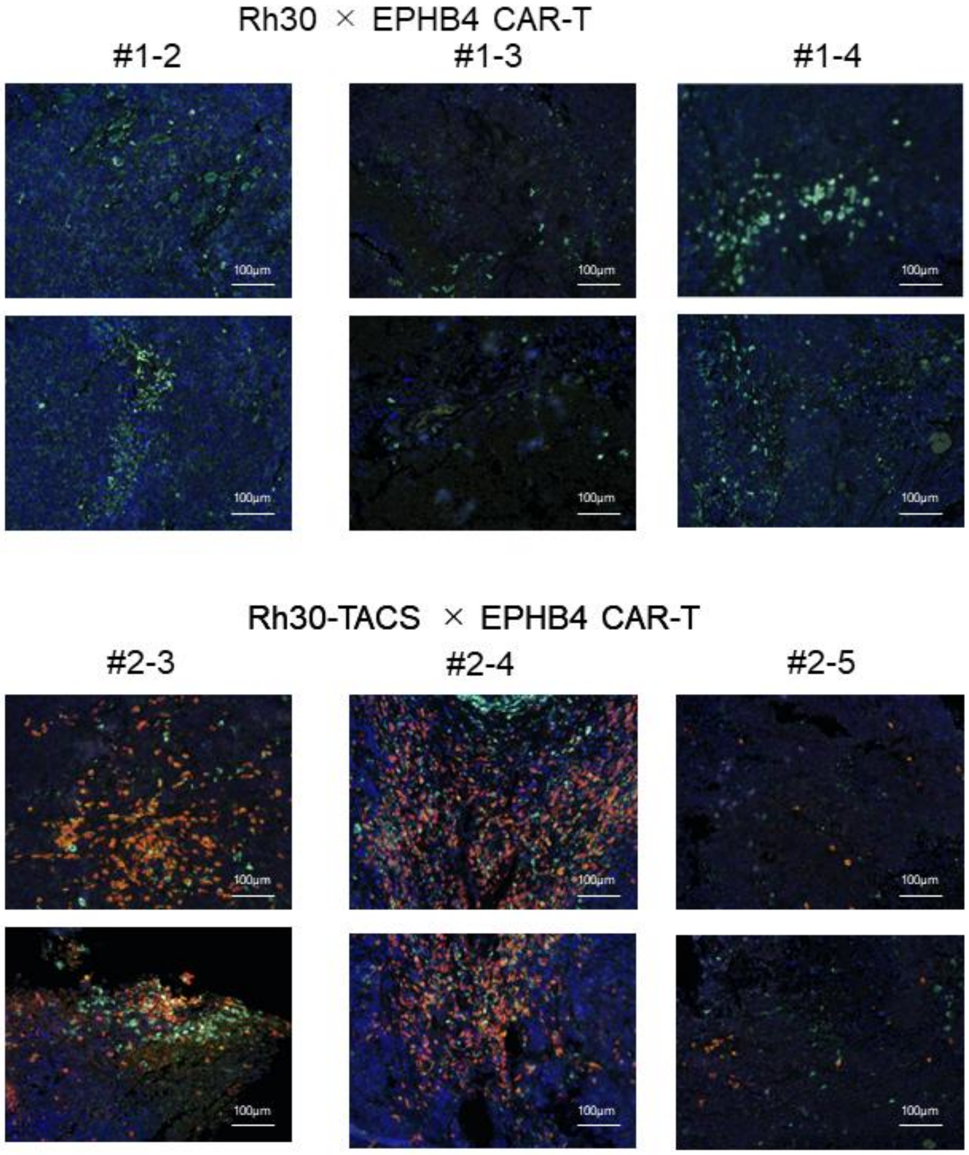

Figure S4. High-magnification (×400) views corresponding to Figure S3, highlighting regions of immune cell infiltration.

Multiplex immunofluorescence imaging and analysis of tumor-infiltrating immune cells in Rh30- and Rh30-TACS–bearing mice treated with EPHB4 CAR-T cells (n = 3 per group). High-magnification (×400) views corresponding to Figure S3 highlight regions of immune cell infiltration. Primary antibodies included anti-CD3 (red), anti-CD8 (yellow), anti–PD-1 (orange), and anti-CD68 (green). Nuclei were counterstained with DAPI (blue). Images corresponding to mouse #1-2 and #2-3 were reused from Figure 2F.

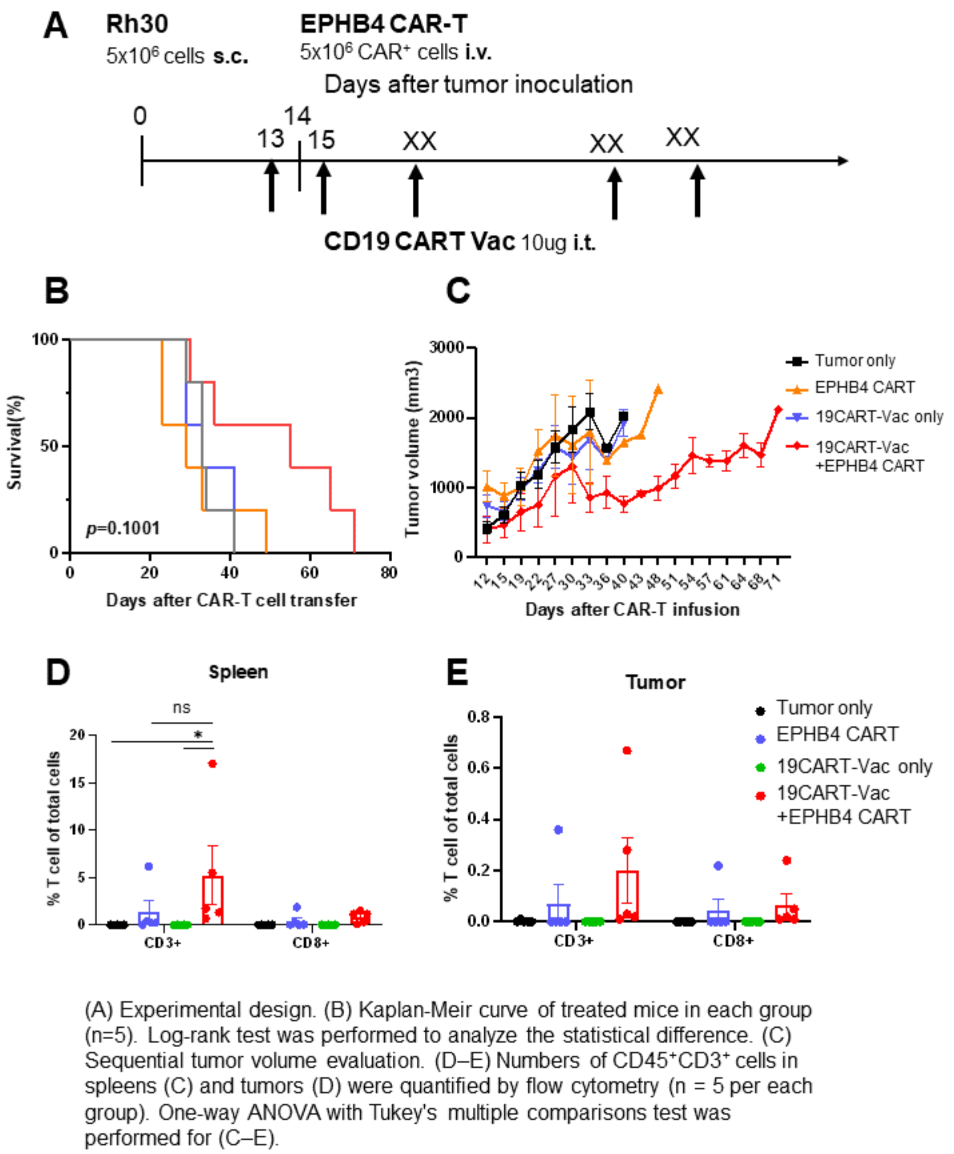

Figure S5. CART-Vac with an irrelevant TA partially enhances the antitumor effects of EPHB4 CAR-T cells.

(A) Experimental design. (B) Kaplan-Meier curve of treated mice in each group (n=5). Log-rank test was performed to analyze the statistical difference. (C) Sequential tumor volume evaluation. (D–E) Numbers of $CD45^{+}CD3^{+}$ cells in spleens (C) and tumors (D) were quantified by flow cytometry (n = 5 per each group). One-way ANOVA with Tukey's multiple comparisons test was performed for (C–E).

Table S1. Antibodies Used for Flow cytometry.

Name	clone	Product#	Company
PE anti-hEphB4	395810	FAB3038P	R&D SYSTEMS
PE anti-CD19	HIB19	302208	BioLegend
PE anti-CD80	2D10	305208	BioLegend
APC anti-CD137L(4-1BBL)	5F4	311506	BioLegend
Goat anti-Ephrin B2	Polyclonal	AF496	R&D SYSTEMS
PE anti-goat IgG Fc	Polyclonal	F0107	R&D SYSTEMS
APC anti-CD3	BW264/56	130-113-125	Miltenyi Biotec
PE anti-CD19CAR FMC63 idiotype	REA1297	130-127-342	Miltenyi Biotec
APC anti-CD3	REA613	130-113-135	Miltenyi Biotec
Alexa Flour 700 anti-CD45	HI30	304024	BioLegend
Brilliant Violet 605 anti-CD3	SK7	344836	BioLegend
Brilliant Violet 785 anti-CD4	RPA-T4	300554	BioLegend
Pacific Blue anti-CD8	B9.11	A82791	Beckman Coulter
APC anti-CD279 (PD-1)	EH12.2H7	329908	BioLegend

Table S2. Antibodies Used for Immunohistochemistry.

Name	clone	source	Product#	Company	dilution	retrieval
anti-EphB4	H-10	Mouse	sc-365510	Santa Cruz	1:50	EDTA(pH9)
anti-CD80	EPR1157(2)	Rabbit	ab134120	Abcam	1:500	EDTA(pH9)
anti-4-1BBL	CD137L/1547	Mouse	ab223160	Abcam	1:100	EDTA(pH9)
anti-CD3	SP7	Rabbit	ab16669	Abcam	1:100	Citrate(pH6)
anti-CD8α	C8/144B	Mouse	ab17147	Abcam	1:1000	EDTA(pH9)
anti-PD-1	EPR4877(2)	Rabbit	ab137132	Abcam	1:250	EDTA(pH9)
anti-CD68	Polyclonal	Rabbit	ab125212	Abcam	1:200	Citrate(pH6)
